# Case report: Metastatic pancreatic neuroendocrine tumour associated with portal vein thrombosis; successful management with subsequent pregnancies

**DOI:** 10.3389/fendo.2023.1095815

**Published:** 2023-02-27

**Authors:** Lívia Sira, Noémi Zsíros, László Bidiga, Sándor Barna, Zsolt Kanyári, Edit B. Nagy, Nicolas Guillaume, Damian Wild, Katalin Rázsó, Szilvia Andó, István Balogh, Endre V. Nagy, Zoltán Balogh

**Affiliations:** ^1^ Division of Endocrinology, Department of Internal Medicine, Faculty of Medicine, University of Debrecen, Debrecen, Hungary; ^2^ Department of Pathology, Faculty of Medicine, University of Debrecen, Debrecen, Hungary; ^3^ Department of Nuclear Medicine, Faculty of Medicine, University of Debrecen, Debrecen, Hungary; ^4^ Department of Surgery, Faculty of Medicine, University of Debrecen, Debrecen, Hungary; ^5^ Department of Radiology, Faculty of Medicine, University of Debrecen, Debrecen, Hungary; ^6^ Clinic of Radiology and Nuclear Medicine, University of Basel Hospital, Basel, Switzerland; ^7^ Division of Haematology, Department of Medicine, Faculty of Medicine, University of Debrecen, Debrecen, Hungary; ^8^ Division of Clinical Genetics, Department of Laboratory Medicine, Faculty of Medicine, University of Debrecen, Debrecen, Hungary

**Keywords:** splanchnic vein thrombosis, metastatic pancreatic neuroendocrine tumour, pregnancy, thrombocytosis, complete remission

## Abstract

**Background:**

Splanchnic vein thrombosis due to co-existing metastatic pancreatic neuroendocrine tumour (pNET) and JAK2V617F mutation is a rare condition.

**Case report:**

Here we present a case of a young woman with complete remission of a non-functioning grade 2 pNET with unresectable liver metastases, coexisting with JAK2V617F mutation. Splenectomy and distal pancreatectomy were performed. Neither surgical removal, nor radiofrequency ablation of the liver metastases was possible. Therefore, somatostatin analogue (SSA) and enoxaparine were started. Peptide receptor radionuclide therapy (PRRT) was given in 3 cycles 6-8 weeks apart. Genetic testing revealed no multiple endocrine neoplasia type 1 (MEN-1) gene mutations. After shared decision making with the patient, she gave birth to two healthy children, currently 2 and 4 years old. On pregnancy confirmation, SSA treatment was interrupted and resumed after each delivery. Ten years after the diagnosis of pNET, no tumour is detectable by MRI or somatostatin receptor scintigraphy. PRRT followed by continuous SSA therapy, interrupted only during pregnancies, resulted in complete remission and enabled the patient to complete two successful pregnancies.

## Introduction

Splanchnic vein thrombosis (SVT), including portal vein, splenic vein, mesenteric vein thrombosis and the Budd-Chiari syndrome, is a manifestation of unusual-site venous thromboembolism. All may present with uncharacteristic epigastrial pain. Predisposing factors are liver cirrhosis, myeloproliferative neoplasms, liver and pancreatic malignancies, factor V Leiden mutation, protein C or protein S deficiency, microparticles, JAK2V617F mutation, and methylenetetrahydrofolate reductase (MTHFR) C677T and A1298C polymorphisms. The association between solid cancers and SVT can be explained by cancer-related haemostatic system alterations. In addition, there are published data on the association of MEN1 syndrome with portal or splenic vein thrombosis (1, 2).

Essential thrombocythemia (ET) is characterized by thrombocytosis and thromboembolic complications. More than half of ET patients are JAK2V617F mutation-positive. According to our present knowledge, JAK2 mutation has been an independent factor for thromboembolic events; however, the precise mechanism remains unknown (3). Most ET patients enjoy a near-normal life expectancy (4).

Up to 2% of all pancreatic tumours are neuroendocrine tumours (5) which can be classified into functioning and non-functioning subtypes. Hormone-producing pancreatic neuroendocrine tumours (pNET) produce a characteristic set of symptoms, therefore they can be detected earlier. Symptoms correspond to the hormone overproduction, and may be related to insulin, gastrin, glucagon, somatostatin, serotonin, or vasoactive intestinal polypeptide. The diagnosis of non-hormone-producing tumours is often delayed for years; 60 to 90% of pNETs are non-functioning and asymptomatic (6).

In Europe and in the USA the incidence of gastroenteropancreatic (GEP) tumours has increased over the last four decades from 0.27 to 1.00 per 100,000 individuals (7). This increase may be due to higher awareness, improved classifications and better diagnostic methods (8). Unfortunately, despite improvement in diagnostic tools, more than half of patients present with distant metastases, mainly in the liver and in lymph nodes (9); these metastases have significant prognostic value in pNET patients (10). For the last two decades, the classification and grading systems of pNETs, which are based on proliferative activity (Ki-67) and mitotic count (MIB-1 proliferation index), have repeatedly been modified (11, 12). Ninety-five percent of the pNETs are sporadic and 5% of them are inherited. The latter is the second most common component of MEN-1 and the leading cause of death among patients with MEN-1 (13).

## Case presentation

Written informed consent for publication of the clinical data and images was obtained from the patient.

In January 2011, a 25-year-old woman was admitted to our gastroenterology unit due to recurrent epigastric pain. Gastroscopy confirmed reflux esophagitis, gastric varices and gastritis. Contrast-enhanced abdominal computed tomography (CT) demonstrated splenomegaly, splenic infarction, portal and splenic vein thrombosis, and an 85x50 mm inhomogeneous necrotic lesion located between the stomach, spleen and left kidney, adjacent to the pancreas. Color Doppler ultrasonography of the abdominal vessels showed massive portal and splenic vein thrombosis. Anticoagulation treatment was immediately started. Low-molecular-weight heparin was followed by warfarin, later switched to rivaroxaban. In April 2011 bone marrow immunophenotyping by flow cytometry did not find any abnormality and chromosome analysis did not detect clonal chromosome aberration. The enlarged spleen was considered a consequence of splenic vein thrombosis. The inhomogeneous necrotic lesion in the left abdomen was assumed to be caused by thrombus in the splenic vein therefore biopsy was not performed. During regular control visits white blood cell count and hemoglobin levels were in the normal range.

While JAK2 V617F mutation was detected, the presence of FV Leiden mutation, prothrombin 20210A allele, lupus anticoagulant, antiphospholipid syndrome and the deficiency of antithrombin-III, protein C or protein S were excluded. The patient neither used oral contraceptives nor was a smoker.

**Figure 1:**
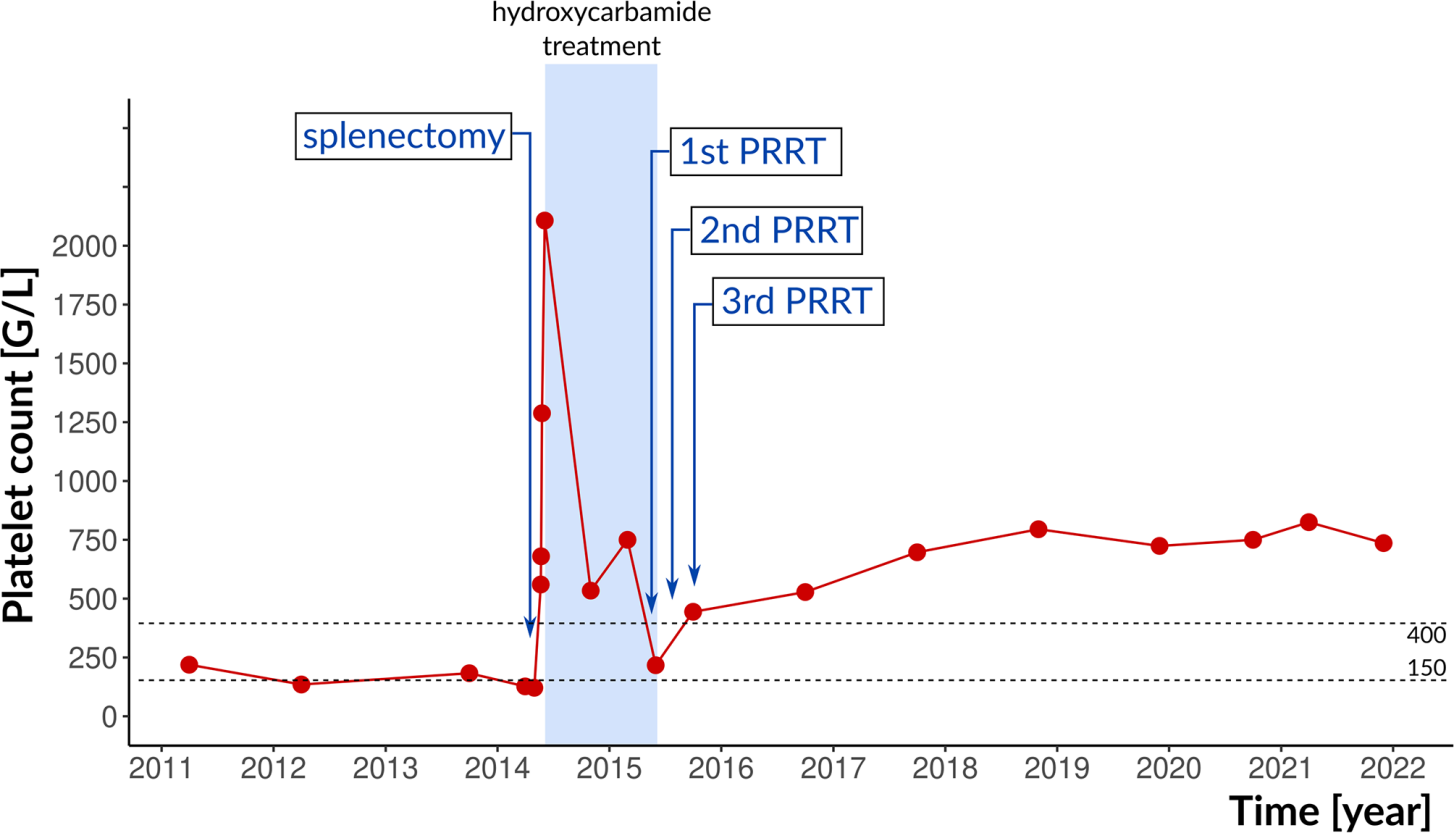
Therapeutic interventions and platelet counts. The reference range is indicated by dotted lines. PRRT, peptide radioreceptor therapy.

Three years later, in 2014, while on rivaroxaban, the patient was admitted to the Emergency Department for upper left abdominal pain. Physical examination showed extreme splenomegaly, without hepatomegaly. Abdominal ultrasound found splenomegaly of 7.5 x 23 cm in addition to the previously diagnosed portal vein thrombosis and dilatation of splenic vein. Platelet count was 121 G/L (**Figure 1**). As the rapid development of painful splenomegaly may have been due to myelofibrosis, bone marrow biopsy was performed. High number of giant megakaryocytes with staghorn-like nuclei, evenly distributed in the specimen, were described, and the possibility of ET was raised by the histopathologist, albeit the peripheral blood platelet count was normal. The granulocyte and erythrocyte lineages were intact and no fibrosis was present. ET could not be proven according to the 2008 WHO diagnostic criteria, as one of the four criterion, namely sustained platelet count >450 G/L was not met (14).

Splenectomy was performed due to the increased risk of splenic rupture. Histopathology of the spleen revealed numerous necrotic neuroendocrine tumour foci with vascular and splenic vein invasion. By immunohistochemistry, tumour cells were neuron-specific enolase (NSE), chromogranin, cytokeratin 7 (CK7) and pan-CK positive. The MIB-1 proliferation index was 5%. There were no symptoms of hormone overproduction.

After splenectomy, the platelet count increased to 2107 G/L, mainly due to reactive thrombocytosis but the possibility of ET could not be clearly ruled out. Interferon therapy was not covered by insurance in Hungary, therefore, hydroxycarbamide was applied for rapid cytoreduction. At that time, the patient was not yet married and did not plan pregnancy. Enoxaparine and allopurinol were added. Platelet count gradually decreased to the normal range within a year.

**Figure 2:**
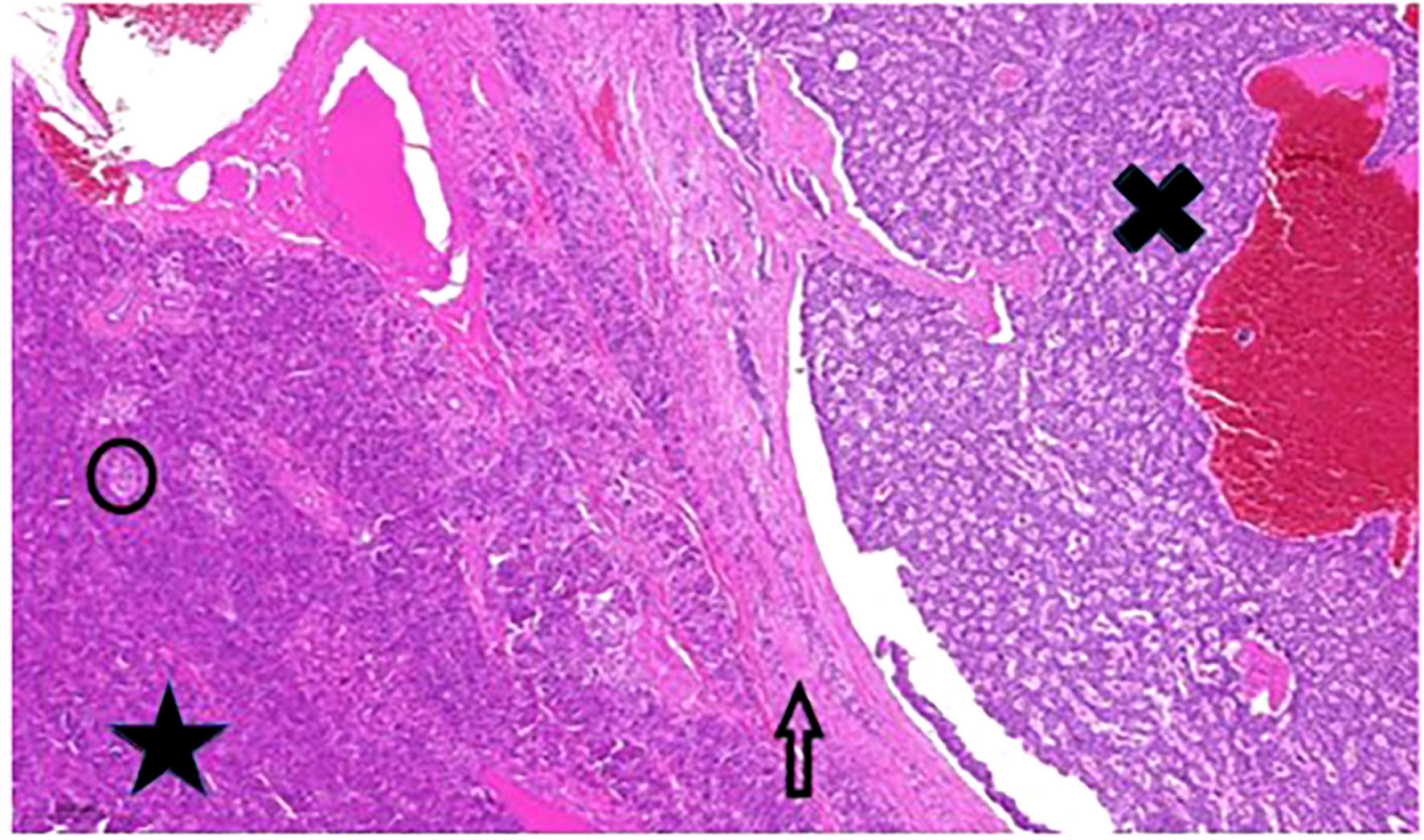
Pancreatic tumour histology. Large well-circumscribed tumour (black X) adjacent to normal pancreas tissue (black star). The arrow points to the fibrotic capsule-like pushing border zone”. The black circle contains one of the Langerhans islands. Hematoxylin and eosin stain, 200x magnification.

^18^F-fluorodeoxyglucose positron emission tomography/computed tomography (FDG PET/CT) demonstrated a 13 cm, irregular soft tissue mass with intensive metabolism in the pancreas. Serum chromogranine-A (CgA) level was >1150 µg/L (reference range: 20-100 µg/L). Somatostatin receptor scintigraphy (SRS) single-photon emission computed tomography/computed tomography (SPECT/CT) using 400MBq ^99m^Tc-EDDA/HYNIC-Tyr3-Octreotide (Tektrotyd, Polatom) showed a 10 cm, irregular, multifocal, highly SRS positive lesion in the splenic bed which could not been separated from the tail of pancreas and stomach wall. Moreover, two focal accumulations with diameters 15 mm and 12 mm were observed in segment VII of the liver. For tumour staging, abdominal CT was performed; the 11 cm diameter lesion in the left hypochondrium spread to nearby organs, and several lesions with diameters between 8-17 mm in segments VII and VIII of the liver were detected. The abdominal lesion was surgically removed by distal pancreatectomy (**Figure2**). Histological examination confirmed G2 pancreatic neuroendocrine tumour according to the World Health Organization (WHO) grading system for pNETs in 2010 (11), with MIB-1 proliferation index of 6%. Immunohistochemistry showed tumour cells with chromogranin+/CDX2+/CK7+/synaptophysin-/CD56-/TTE1- expression pattern. The liver metastases could not be surgically removed, and radiofrequency ablation was hindered by the proximity of the diaphragm.

After pancreas surgery, lanreotide 120 mg every four weeks was started; serum CgA level decreased to 156.4 µg/L (reference range 20-100 µg/L). After one year, control SRS showed 4.4 cm and 2.3 cm focal lesions in segment VII of the liver with marked radiopharmaceutical accumulation. In 2015, peptide radioreceptor therapy (PRRT) was performed: DOTA-d-Phe (1)-Tyr (3)-octreotide (DOTATOC) therapy was given in 3 cycles 6-8 weeks apart (180 mCi of ^90^Y-DOTATOC followed by 200 mCi of ^177^Lu-DOTATOC and 200 mCi of ^177^Lu-DOTATOC). Treatments were well tolerated. The liver and kidney function remained normal. The elevated platelet count normalized after the first treatment; hydroxycarbamide treatment was stopped. One year after PRRT, in December 2016, abdominal CT, liver magnetic resonance imaging (MRI) and octreoscan SPECT/CT showed significant regression of the liver metastases without tumour recurrence in the pancreatic region. Over the next three years, platelet counts were in the 217 to 679 G/L range without any specific treatment, followed by a rise to the current 736 G/L (**Figure 1**). Lanreotide and rivaroxaban were continued.

**Figure 3:**
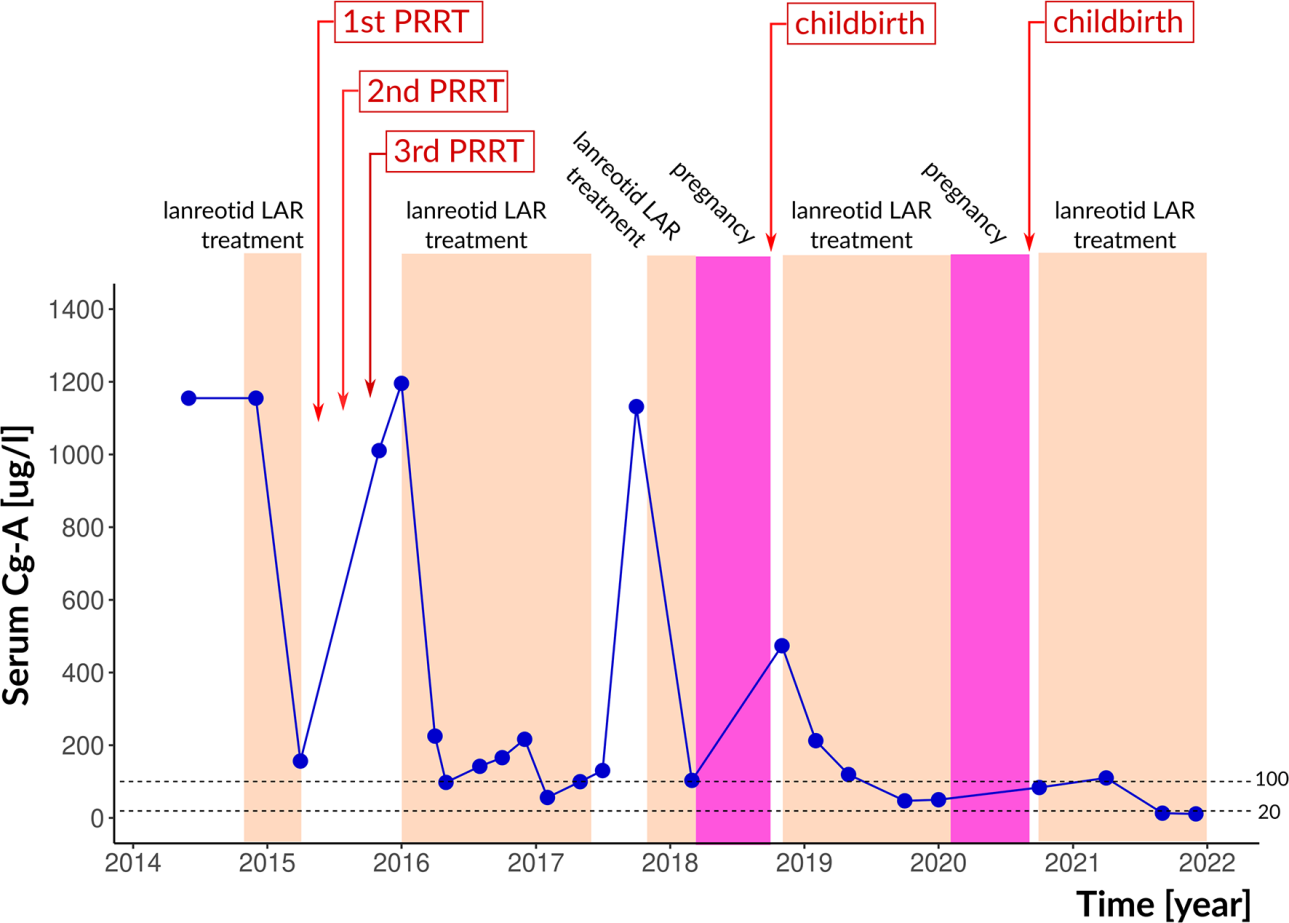
Therapeutic interventions, pregnancies and serum chromogranin-A levels. The reference range is indicated by dotted lines. Cg-A, chromogranin-A.

**Figure 4:**
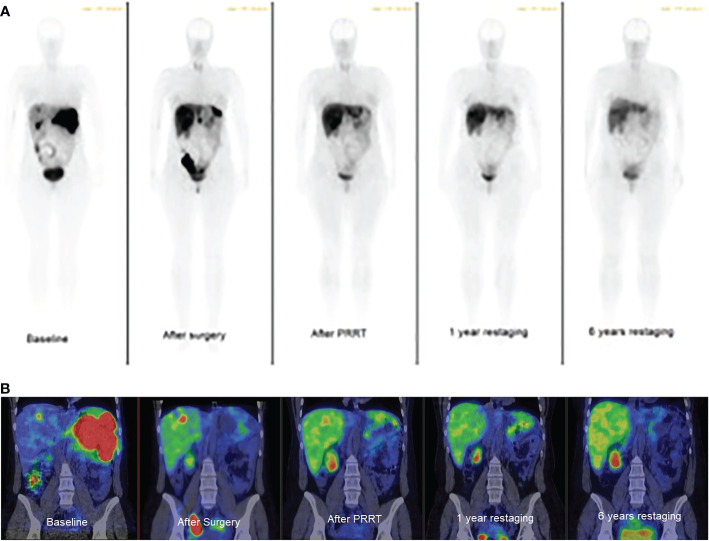
Whole-body somatostatin receptor imaging. **(A)**: planar gamma camera images, anterior view, **(B)**: abdominal SPECT/CT images, coronal view. Baseline: multiple spleen and focal liver metastases. After surgery: focal liver and peritoneal oligometastases. After PRRT: focal liver metastasis. 1 year restaging: focal liver metastasis, 6 years restaging: no visible metastasis. Metastases are black on planar and red on SPECT/CT images.

Later, in 2017, lanreotide was temporarily interrupted because of planned pregnancy. While on lanreotide, CgA levels were between 83.7 and 225.1 µg/L; it increased to 1137.7 µg/L when lanreotide was discontinued. After shared decision making with the patient, lanreotide was resumed and continued until pregnancy occurred. In March 2018, a 7 weeks pregnancy was confirmed. SSA treatment was immediately discontinued and rivaroxaban was switched to LMWH. In October 2018, the patient gave birth to a healthy daughter. One month after delivery, lanreotid treatment was resumed. Serum CgA level returned to the normal range and remained low during the three-monthly controls (**Figure 3**). In February 2020, a second pregnancy was confirmed, therefore SSA treatment was discontinued, and rivaroxaban was switched to LMWH again. In September 2020, she gave birth to her second healthy daughter. After delivery, no metastasis was detected by MRI in the liver, and neither SRS (**Figure 4**) nor chest and abdominal CT did show metastasis in the lung or abdominal organs. Serum CgA level was in the reference range before the post-pregnancy reinstitution of SSA therapy. Complete remission was achieved.

Screening tests for MEN-1, including the levels of serum calcium, phosphorus, and pituitary hormone levels were in the normal range. Family history of the patient was unremarkable. Whole Exome Sequencing (WES) was performed on the Illumina NextSeq 500 sequencer system in 2 × 150 cycle paired-end mode. A Twist Library Preparation EF kit (Twist Bioscience) was used for library preparation. Raw data were aligned to the hg38 reference genome using the NextGene software (SoftGenetics). The test targets all protein coding exons and exon-intron boundaries (± 20 bps). The analysis was restricted to the following genes: MEN1, VHL, NF1, TSC1, TSC2, MUTYH, BRCA2, CHEK2. No causative variant was identified.

## Discussion

The presented case is instructive in several ways. First, paraneoplasia has to be considered in case of splanchnic vein thrombosis and detailed tumour search is warranted especially in young patients. Second, diagnosis of non-functioning neuroendocrine tumours may delay by several years due to the lack of specific clinical symptoms. Third, systemic therapies such as SSAs and chemotherapy, and targeted therapy using PRRT are therapeutic options for patients with advanced metastatic or inoperable pNET (15–17).

SSAs exert antiproliferative and antisecretory effects. In addition, activation of somatostatin receptor-2 and -3 also have proapoptotic effects (16, 17). In the PROMID study the enrolled 85 patients with G1 advanced midgut NET, octreotide LAR every 4 weeks (vs. placebo) had significant progression-free survival (PFS) improvement (14.3 vs. 6 months, HR 0.34, p=0.000072) (18). The CLARINET trial enrolled 204 patients with advanced non-functioning GEP-NETs with a Ki-67 index <10% and a positive somatostatin receptor scintigraphy; PFS significantly increased in patients treated with lanreotide as compared to placebo (HR=0.47, p=0.0002) (19).

PRRT is suitable for management of advanced, inoperable NETs. ^90^Yttrium or ^177^Lutetium is bound to SSA *via* a chelator and SSA directs the complex to NET cells expressing somatostatin receptors in their surface. In the NETTER-1 trial the results of the interim analysis suggested longer progression-free survival and a higher response rate with ^177^Lu-DOTATATE than with high-dose octreotide LAR (20). Data on overall survival (OS) at the 5 year follow-up have been published; ^177^Lu-DOTATE treatment did not significantly improve median OS vs. high-dose long-acting octreotide-LAR alone. Albeit survival difference did not reach statistical significance, the 11.7 month advantage in median OS with ^177^Lu-DOTATE treatment might be considered clinically relevant (21). The OCLURANDOM trial was designed to evaluate the efficacy of ^177^Lu-DOTATATE vs. sunitinib in patients with SRS positive unresectable progressive advanced pancreatic NETs; median PFS was 20.7 months (90% CI; 17.2–23.7) in the ^177^Lu-DOTATATE arm *vs*.11.0 months (90% CI; 8.8–12.4) in the sunitinib arm (22).

A retrospective study demonstrated favourable response and long-term outcome in patients with metastatic G1/G2 GEP NET after ^177^Lu-octreotate PRRT (23). Our patient did benefit from PRRT: the liver metastases regressed. PRRT is known to cause bone marrow suppression for several years, and indeed, her platelet count markedly decreased after ^90^Y-DOTATOC treatment. Although post-splenectomy reactive thrombocytosis was evident, it was tempting to raise the possibility of ET based on the JAK2 mutation, bone marrow biopsy, and the long-lasting thrombocytosis during the following 6 years. Indeed, splenectomy may unmask JAK2 positive ET (24). However, in the present case, we cannot draw a unanimous conclusion about this.

Serum CgA level was a reliable tumour marker during follow-up. CgA is considered one of the best-described laboratory biomarkers of NETs with a sensitivity of 66%, specificity of 95%, and overall accuracy of 71% (25). After learning the related risks, our patient has chosen to become pregnant. SSA was not stopped until pregnancy was proven. Two subsequent uneventful pregnancies suggest that lanreotide may be safe, if uncontrolled CgA elevation prevents its preventive discontinuation. Two cases of non-functioning, Grade 2 pNET cases diagnosed during pregnancy were reported to undergo successful surgical resection of the tumour in the second trimester of pregnancy (26). A G2 functioning gastrinoma with liver metastasis was reported to dedifferentiate after pregnancy to a poorly differentiated Grade 3 large-cell neuroendocrine cancer (27). PRRT before pregnancy may have contributed to the lack of such phenomenon in our patient.

SSA and PRRT are options for patients who are unsuitable for liver-directed debulking treatment. Although complete remission is rare after PRRT of patients with inoperable or metastatic pNET, there are some case reports about complete remission (28, 29). The exact place of PRRT in the treatment of G1-G2 pNET is not entirely clear. According to European Society for Medical Oncology (ESMO) guideline (30) SSAs is recommended as first-line therapy for tumour growth control in advanced, slowly-growing SSTR-positive gastrointestinal and pNETs with Ki-67 up to 10%. According to the European Neuroendocrine Tumor Society (ENETS) consensus guideline (31) ^177^Lu-PRRT is recommended for treatment of metastatic or inoperable well-differentiated (Grade 1 or 2) NET after failure of medical therapy including SSA, chemotherapy, or novel targeted drugs (sunitinib or everolimus). It remains unclear if, and how long SSA should be continued after PRRT as a maintenance therapy. Opinions favoring the early consideration of PRRT are accumulating (31, 32).

There are several limitations of our case report. Firstly, a single case is presented. Second, we were not able to decide if the persistent high platelet count was due to the splenectomy or a prodromal stage of ET. The strength is the 11-year follow-up of the patient, including her two successful pregnancies. The patient continues to be under regular imaging, laboratory and hematological control. Both children are healthy and followed up by the pediatrician.

Conclusion

Three cycles of PRRT followed by continuous SSA therapy, interrupted only during pregnancies, enabled the patient to complete two successful pregnancies while complete remission was also achieved. Paraneoplasia has to be considered in case of splanchnic vein thrombosis and detailed tumour search is warranted especially in young patients. The diagnosis of non-functioning neuroendocrine tumours may delay by several years due to the lack of specific clinical symptoms.

## Data availability statement

The data presented in the study are deposited in the NCBI Sequence Read Archive (SRA) repository, accession number PRJNA902518 (BioSample: SAMN31762934, SRA: SRR22317887), https://www.ncbi.nlm.nih.gov/bioproject/PRJNA902518.

## Ethics statement

Ethical review and approval was not required for the study on human participants in accordance with the local legislation and institutional requirements. The patients/participants provided their written informed consent to participate in this study. Written informed consent for publication of the clinical data and images was obtained from the patient.

## Author contributions

LS, NZ and KR cared for the patient. LS, ZB and EVN participated in the conceptualisation and manuscript preparation. LB made histopathological examination. SB performed somatostatin receptor scintigraphy. ZK performed the surgical resections. EBN evaluated the CT and MR images. NG and DW performed PRRT. SA and IB made the genetic testing. ZB and EVN finalized the manuscript. All authors contributed to the article and approved the submitted version.
